# Hidden Population Structure and Cross-species Transmission of Whipworms (*Trichuris* sp.) in Humans and Non-human Primates in Uganda

**DOI:** 10.1371/journal.pntd.0003256

**Published:** 2014-10-23

**Authors:** Ria R. Ghai, Noah D. Simons, Colin A. Chapman, Patrick A. Omeja, T. Jonathan Davies, Nelson Ting, Tony L. Goldberg

**Affiliations:** 1 Department of Biology, McGill University, Montreal, Quebec, Canada; 2 Department of Anthropology, University of Oregon, Eugene, Oregon, United States of America; 3 Department of Anthropology and McGill School of Environment, Montreal, Quebec, Canada, and Wildlife Conservation Society, Bronx, New York, New York, United States of America; 4 Makerere University Biological Field Station, Fort Portal, Uganda; 5 Institute for Ecology and Evolution, University of Oregon, Eugene, Oregon, United States of America; 6 Department of Pathobiological Sciences and Global Health Institute, University of Wisconsin-Madison, Madison, Wisconsin, United States of America; University of Melbourne, Australia

## Abstract

**Background:**

Whipworms (*Trichuris* sp.) are a globally distributed genus of parasitic helminths that infect a diversity of mammalian hosts. Molecular methods have successfully resolved porcine whipworm, *Trichuris suis*, from primate whipworm, *T. trichiura*. However, it remains unclear whether *T. trichiura* is a multi-host parasite capable of infecting a wide taxonomic breadth of primate hosts or a complex of host specific parasites that infect one or two closely related hosts.

**Methods and Findings:**

We examined the phylogenetic structure of whipworms in a multi-species community of non-human primates and humans in Western Uganda, using both traditional microscopy and molecular methods. A newly developed nested polymerase chain reaction (PCR) method applied to non-invasively collected fecal samples detected *Trichuris* with 100% sensitivity and 97% specificity relative to microscopy. Infection rates varied significantly among host species, from 13.3% in chimpanzees (*Pan troglodytes*) to 88.9% in olive baboons (*Papio anubis*). Phylogenetic analyses based on nucleotide sequences of the *Trichuris* internal transcribed spacer regions 1 and 2 of ribosomal DNA revealed three co-circulating *Trichuris* groups. Notably, one group was detected only in humans, while another infected all screened host species, indicating that whipworms from this group are transmitted among wild primates and humans.

**Conclusions and Significance:**

Our results suggest that the host range of *Trichuris* varies by taxonomic group, with some groups showing host specificity, and others showing host generality. In particular, one *Trichuris* taxon should be considered a multi-host pathogen that is capable of infecting wild primates and humans. This challenges past assumptions about the host specificity of this and similar helminth parasites and raises concerns about animal and human health.

## Author Summary

Whipworms are a group of gastrointestinal worms that are both common and globally distributed. These parasites are known to stunt development, especially in school-aged children, and therefore hinder economic, social, and intellectual growth. Unfortunately, research on whipworms has lagged behind its effects, at least in part because this parasite infects the world's poorest populations. Currently, a single species of whipworm is assumed capable of infecting both humans and non-human primates. In this study, we tested this assumption by collecting fecal samples from humans and overlapping non-human primate populations containing monkeys and chimpanzees in Western Uganda. Using molecular analyses, we examined patterns of genetic similarity between human and nonhuman primate whipworms. We identified three genetically distinct groups of whipworms that could not be distinguished by microscopic examination of their eggs. One of these groups was found in all nine species of primates examined, including humans. These findings suggest that some varieties of whipworms are indeed transmissible between humans and non-human primates, which raises concerns for both human health and conservation.

## Introduction

Parasites that infect multiple host species are of particular concern because they are more likely to emerge than single-host parasites [1]–[4]. Moreover, multi-host parasites are difficult to control because reservoir hosts may serve as sources of re-infection for other populations in which the parasite has been eliminated [5]–[7]. A number of ecological and evolutionary factors influence the range of hosts that a parasite can infect (host specificity). Multi-host parasites of non-human primates (hereafter primates) have come under particular scrutiny, because physiological similarity (due to relatedness) between primates and humans increases the potential for zoonotic transmission. Indeed, phylogenetic relatedness between primate hosts is a stronger predictor of parasite sharing than geographic overlap [8]. Despite the probability of parasite sharing between primates and humans, only 20% of primate helminths (parasitic worms) are thought to infect humans [9]. Conversely, half of all primate helminths are thought to be specific to a single host species [9], [10]. These observations suggest that, compared to other taxonomic groups of parasites, helminths have a lesser propensity for zoonotic transmission, perhaps because of their physical complexity, indirect life cycles, and long generation times [5], [11].

Here we examine the host specificity of the whipworm genus *Trichuris*, a soil-transmitted helminth with a global distribution [12]. *Trichuris trichiura* is estimated to affect approximately 600 million people worldwide [13], [14], causing physical and mental growth retardation in children [12], [15]. *Trichuris* infection results from ingestion of embryonated eggs shed into food, water, and soil [15]. Following ingestion, first-stage larva (L1s) hatch and move through the gastrointestinal tract where they develop in the caecum, molt into adults, and tunnel into the mucosa of the large intestine. After mating, female whipworms release eggs into feces. Eggs typically become infective after 20 days or more in the environment, where they are tolerant to desiccation and temperature extremes [16]–[20].

Currently, the *Trichuris* genus contains more than 20 described species that are generally specific to taxonomic groups of hosts [18]. Traditional parasitological research on the genus has focused on differentiating *Trichuris trichiura*, found in humans and primates, from *Trichuris suis*, found in pigs [21]–[25]. Morphologically, these two species are similar, and previous attempts to distinguish them based on variation in reproductive organ morphology were inconclusive because phenotypic plasticity could not be distinguished from genotypic differences [26]. The unsuitability of morphological characteristics for resolving differences between *T. trichiura* and *T. suis* made molecular methods a promising approach. Sequences from the internal transcribed spacer regions 1 and 2 (ribosomal DNA) from primate and porcine hosts suggest that *T. trichiura* and *T. suis* are two closely related but separate species [22], a conclusion further supported by subsequent analyses of β tubulin gene sequences [23].

Morphological studies of *Trichuris* isolated from primates and humans conclude that the species infecting these hosts is the same, despite slight morphological variations that are distinguishable using scanning electron microscopy [21]. These results suggest that both primates and humans are infected with *T. trichiura*, which is capable of freely switching between primate and human hosts. Perhaps as a result of these findings, DNA sequences isolated from both primate and human hosts have been assumed to be *T. trichiura* by virtue of the host alone, and without the taxonomic scrutiny required to identify the parasite to species level. An empirical test of the assumption that primate and human *Trichuris* are identical used molecular methods to sequence DNA from *Trichuris* adults isolated from chacma baboons (*Papio ursinus*) and humans. Results revealed two distinct lineages of *Trichuris* in baboons [27]. The authors concluded that both lineages were transmissible between humans and baboons, and that *T. trichiura*, while perhaps not a single lineage, is a zoonotic parasite. Transmission between humans and primates is additionally supported by a molecular study of both β tubulin and ITS 2 gene regions isolated from both humans and baboons (*Papio anubis, P. hamadryas*), where no genetic differentiation between host species was found [28]. In contrast, work on both ribosomal DNA and complete mitochondrial genome sequences has found evidence of host specificity within the *Trichuris trichiura* species complex [29]. These results led to the suggestion that *Trichuris trichiura* is not a single multi-host parasite, but rather a complex of host-specific lineages, each infecting distinct taxonomic groups of primates [29]. This suggestion is supported by molecular data from a small number of studies in non-human primate taxa [23], [24], [29]–[32].

In this study, we examine the phylogenetic structure of *Trichuris* in a host community comprised of wild primates and a nearby human population. Our study is based in and around Kibale National Park, Uganda, where *Trichuris* is known to infect several species [33]–[37]. Humans and primates in this region frequently overlap. For example, several species of primates raid crops, and people often enter the park to extract resources such as wood, food, and traditional medicines [38]–[41]. People and primates are exposed to the same physical environment during such events and can even interact directly [42]. Thus, the Kibale ecosystem is useful for examining the host specificity of parasites in a setting where cross-species transmission, including zoonotic transmission, is ecologically possible. Indeed, previous research in Kibale has demonstrated cryptic genetic lineages and cross-species transmission of another soil-transmitted helminth genus of primates and humans, the nodule worm (*Oesophogostomum* spp.) [43]. Our results herein demonstrate that the taxonomy and population structure of *Trichuris* is more complex than previously appreciated. Specifically, we identify cryptic *Trichuris* lineages, of which some infect multiple primate host species, including humans.

## Methods

### Ethics Statement

Prior to data collection, this research protocol was approved by the Uganda National Council for Science and Technology, the Uganda Wildlife Authority, and the Institutional Review Board and Animal Care and Use Committees of McGill University and the University of Wisconsin-Madison. Due to low literacy, a combination of written and oral consent following World Health Organization protocols was obtained from all participants or their parents/guardians. Consent was obtained by trained local field assistants and documented on IRB-approved forms. Samples were collected, processed and shipped according to the guidelines outlined by the Uganda National Council for Science and Technology, the Uganda Wildlife Authority, and the Public Health Agency of Canada.

### Study Site and Collection Methods

Kibale National Park (0°13′-0°41′N, 30°19′-30°32′ E) is a 795 km^2^ mid-altitude rainforest located in Western Uganda. Kibale harbors nine species of diurnal primate that have been the focus of over four decades of research on primate ecology [44]–[47], and infection, including zoonoses [42], [48]–[54]. Kibale is surrounded by a dense human population of up to 600 people/km^2^ [55]. Sample collection occurred in and around Kanyawara, a North Western segment of the park (see Ghai et al. [43]).

*Trichuris* and other gastrointestinal helminths pass their eggs in the feces of their host, which offers an opportunity to conduct molecular analysis non-invasively by isolating DNA directly from parasite eggs. We collected primate fecal samples non-invasively from individuals in habituated primate groups. All primate groups were sampled only once to prevent pseudo-replication of individuals. Fecal samples were collected immediately after defecation and placed in sterile tubes. Seven diurnal monkey species were sampled: black-and-white colobus (*Colobus guereza*), blue monkeys (*Cercopithecus mitis*), grey-cheeked mangabeys (*Lophocebus albigena*), l'hoest monkeys (*Cercopithecus lhoesti*), olive baboons (*Papio anubis*), red colobus (*Procolobus rufomitratus*), and red-tailed guenons (*Cercopithecus ascanius*). Chimpanzee (*Pan troglodytes*) samples were collected from two habituated groups in Kanyanchu, a section of Kibale approximately 15 km from Kanyawara. Human samples were collected from individuals between the ages of 2 and 70 residing in one of three villages within 5 km of the park boundary. Following informed consent, participants were provided with collection materials and instructions, and samples were retrieved within one day for processing.

All samples underwent a procedure to concentrate nematode eggs while removing particles and debris. A modified ethyl acetate sedimentation method using one gram of feces was chosen due to its suitability for field conditions and its efficacy at recovering helminths eggs [56]. Details are provided elsewhere [43]. Samples were collected between May and August 2011.

### Microscopy

We used microscopy to confirm infection status by identifying *Trichuris* eggs. Thin smears of sedimented feces were examined under 10X objective magnification on a Leica DM2500 light microscope. Length, width, color, and contents of eggs were recorded at 40X magnification, and images were captured with an Infinity1 CMOS digital microscope camera and Infinity Camera v.6.2.0 software (Lumenera Corporation, Ottawa, ON, Canada). Samples were considered positive for *Trichuris* when one or more eggs with the characteristic *Trichuris* “lemon” shape were identified. Samples were considered free of *Trichuris* only after the entire sediment was scanned and no *Trichuris* eggs were seen. All samples were examined by the same observer (RRG) to avoid inter-observer bias.

### Molecular Methods

DNA was extracted from 200 µl of sedimented feces preserved in RNAlater nucleotide stabilization solution (Sigma-Aldrich, St. Louis, MO, USA) using a ZR Fecal DNA MiniPrep Kit (Zymo Research Corporation, Irvine, CA, USA), following manufacturer protocols.

The parasite internal transcribed spacer region (ITS) 1 of the ribosomal DNA complex was amplified using polymerase chain reaction (PCR) with newly designed primers that were specific to the genus *Trichuris*. These primers were nested within the 18S (small ribosomal subunit) coding region and the 5.8S non-coding region (see Romstad et al. [57]). Two forward primers (external and internal) were sited within conserved regions of 18S sequences of *T. trichiura* (Genbank accession numbers: AB699091, AB699090, AB699092, GQ352548), *T. suis* (accession no.AY851265), *T. vulpis* (accession no. GQ352558), and *T. muris* (accession no. AF036637). Other enoplean nematodes (*Romanomermis*, accession no. AY146544; *Agamermis*, accession no.DQ628908; *Capillaria*, accession no. EU004822; and *Trichinella*, accession no's. U60231 and AY487254), as well as representative genera likely to occur in Kibale (*Caenorhabditis*, accession no. JN636068; *Strongyloides*, accession no. M84229) were included in primer design alignments to ensure primers were specific to *Trichuris*. The two generated primers were: External_*Trichuris*-1417F (5′-AGGGACCAGCGACACTTTC-3′) and Internal_*Trichuris*-1567F (5′-GTTCTCGTGACTGGGAC-3′).

Reverse primers that were also specific for the genus *Trichuris* were designed in a similar manner, using aligned 5.8S sequences from *T. trichiura* (accession no's. GQ301555, GQ301554, KC877992), *T. suis* (accession no's. JF690951, AM993015), *T*. sp (accession no's. JF690940-JF690952, HQ844233), *T. ovis* (accession no. JX218218), *T. muris* (accession no. FN543201), *T. arvicolae* (accession no. FR849687), and *T. discolor* (accession no. JX281223). Other enoplean nematodes (*Trichinella*, accession no's. AF342803, KC006431) and representative genera likely to be found in Kibale (*Oesophagostomum*, accession no's. AJ619979 and AB821014; *Strongyloides*, accession no. EF653265; *Xiphinema*, accession no. HM990158) were also included. The reverse primers generated were: ExternalITS1_*Trichuris*-2505R (5′-GAGTGTCACGTCGTTCTTCAAC-3′) and InternalITS1_*Trichuris*-2462R (5′-CTACGAGCCAAGTGATCC-3′). External primers generated amplicons of approximately 1088 bp expected size; internal primers generated amplicons of 895 bp expected size.

The ITS 2 region was amplified using primers nested within the 5.8S non-coding and 28S (large ribosomal subunit) coding regions. The ITS 1 internal reverse primer described above (InternalITS1_*Trichuris*-2462R) was reversed and used as the forward external primer (ExternalITS2_*Trichuris*-2462F: 5′-GGATCACTTGGCTCGTAG-3′). The internal primer, InternalITS2_*Trichuris*-2560F (5′-CTTGAATACTTTGAACGCACATTG-3′) was designed using the aligned 5.8S sequences described above and was also specific to the genus *Trichuris*. A previously published, conserved primer NC2 (5′-TTAGTTTCTTTTCCTCCGCT-3′) was used as the reverse primer in both external and internal reactions [58]. External primers generated amplicons of approximately 584 bp expected size; internal primers generated amplicons of 486 bp expected size.

The efficacy of the protocols designed for amplifying only *Trichuris* ITS 1 and 2 regions was tested using dilutions of a positive control (adult *T. vulpis* isolated by necropsy from an infected canine at Cornell University), and by implementing the protocol on samples known to contain infections with multiple parasite genera. The protocol was found to be 100% accurate at detecting only *Trichuris* even among mixed infections.

ITS 1 external PCR was performed in 25 µL volumes using the FailSafe System (Epicentre Biotenchnologies, Madison, WI, USA). Reactions contained 1X FailSafe PCR PreMix with Buffer C (containing dNTPs and MgCl_2_), 1 Unit of FailSafe Enzyme Mix, 2.5 picomoles of each primer (ExternalITS1_*Trichuris*-1417F and ExternalITS1_*Trichuris*-2505R), and 1 µL of template (extracted DNA from sedimented feces). Reactions were cycled in a Bio-Rad CFX96 thermocycler (Bio-Rad Laboratories, Hercules, CA, USA) with the following temperature profile: 94°C for 60 sec; 40 cycles of 94°C for 60 sec, 61°C for 30 sec, 72°C for 75 sec; and a final extension at 72°C for 10 min. Internal PCR was performed in 25 µL volumes using the DyNAzyme DNA Polymerase Kit (Thermo Scientific, Asheville, NC, USA) with reactions containing 0.5 Units of DyNAzyme I DNA Polymerase, 1X Buffer containing 1.5 mM MgCl_2_, 2.5 picomoles of each primer (InternalITS1_*Trichuris*-1567F and InternalITS1_*Trichuris*-2462R)), 0.5 µL dNTPs, and 1 µL of template (product of the external PCR reaction). Reactions were cycled according to the following temperature profile: 94°C for 60 sec; 35 cycles of 94°C for 30 sec, 55°C for 30 sec, 72°C for 75 sec; and a final extension at 72°C for 10 min.

ITS 2 PCR used the same reagents as the ITS 1 external and internal reactions described above, with external reactions using ExternalITS2_*Trichuris*-2462F and NC2 primers, and internal reactions using InternalITS2_*Trichuris*-2560F and NC2 primers. Both external and internal reactions were cycled according to the following temperature profile: 94°C for 60 sec; 35 cycles of 94°C for 30 sec, 55°C for 30 sec, 72°C for 60 sec; and a final extension at 72°C for 10 min. PCR products were electrophoresed on 1% agarose gels stained with ethidium bromide. Amplicons were excised and purified using the Zymoclean Gel DNA Recovery Kit (Zymo Research Corporation, Irvine, CA, USA) according to the manufacturer's instructions.

ITS 1 and 2 products were Sanger sequenced in both directions using primers InternalITS1_*Trichuris*-1567F and InternalITS1_*Trichuris*-2462R for ITS 1 and InternalITS2_*Trichuris*-2560F and NC2 for ITS 2. Sequencing was performed on ABI 3730xl DNA Analyzers (Applied Biosystems, Grand Island, NY, USA) at the University of Wisconsin-Madison Biotechnology Center DNA Sequencing Facility. Sequences were hand-edited and assembled using Sequencher v. 4.9 (Gene Codes Corporation, Ann Arbor, MI, USA) with reference to published sequences. Generation of unambiguous sequences required repeat PCR and re-sequencing on three occasions. Newly generated sequences were deposited in GenBank, under accession numbers KJ588071-KJ588132 (18S, ITS 1) and KJ588133-KJ588167 (5.8S, ITS 2, 28S); see Supplementary Table S1.

### Analyses

The ratio of *Trichuris* egg length to width was calculated and compared among groups using Kruskal-Wallis tests and Dunn's multiple comparison post-tests in Prism6 (GraphPad Software Inc., La Jolia, CA, USA) to assess shape differences between different groups of *Trichuris*. To compare the diagnostic performance of microscopy with newly designed PCR methods, sensitivity (*i.e.*, the proportion of samples correctly identified as positive by PCR as compared to microscopy) and specificity (*i.e*., the proportion of samples correctly identified as negative by PCR) were calculated using MedCalc v. 12.5.0 (MedCalc Software, Ostend, Belgium). Prevalence of *Trichuris* infection was calculated as the total number of positive samples divided by the total number of samples, with 95% confidence intervals calculated using the modified Wald method [59]. Differences in prevalence among host species were evaluated using Fisher's exact tests implemented in the program Quantitative Parasitology v. 3.0 [60].

Due to the number and varying sizes of indels among DNA sequences, we aligned sequences using webPRANK, a phylogeny-aware progressive alignment tool that has been shown to outperform other methods in indel-rich alignment [61], [62]. Aligned sequences were trimmed to consistent length and missing data were coded as “?” in BioEdit v. 7.2.5 [63]. Samples for which both ITS 1 and 2 were generated were concatenated in Sequence Matrix v. 1.7.8 [64]. All sequences were subjected to Gblocks treatment to remove regions of ambiguous alignment using the following parameters: “Maximum number of contiguous non-conserved positions”  = 100, “Minimum length of a block”  = 4, and “Allowed gap positions”  =  half [65]. Models of sequence evolution for each gene were selected using the MrModelTest v. 2 executable in PAUP* v. 4 [66], [67].

We reconstructed phylogenetic relationships using Bayesian methods and HKY+I (ITS 1) and HKY (ITS 2) models, implemented in MrBayes v. 3.2.2 through the CIPRES Science Gateway [68], [69]. Phylogenetic analyses were conducted on concatenated, Gblocks treated ITS 1 and 2 sequences. Four chains were run for 1×10^7^ MCMC generations, sampling every 1000^th^ generation with a diagnostic frequency of every 5000^th^ generation. MCMC runs continued until a standard deviation of split frequency value of 0.01 was reached. Convergence was confirmed when all substitution model parameters reached a potential scale reduction factor value of 1, and was visually assessed using Tracer v. 1.6. The first 10% of runs were discarded as burn-in and Bayesian posterior probabilities were calculated from the remaining trees.

Genetic divergence among *Trichuris* populations was estimated as percent nucleotide-level sequence identity, calculated as the uncorrected pairwise proportion of nucleotides (*p*-distance) in MEGA v. 5.1 with 1000 bootstrap replicates [70]. Analysis of molecular variance (AMOVA) was used to partition *Trichuris* genetic diversity into within host and between host components [71] in GenAlEx v. 6.5 [72]. Pairwise population differentiation values (PhiPT; an analogue of FST), were also calculated in GenAlEx. To assess the relationship between host phylogeny and parasite phylogeny, mantel tests were used to compare pairwise distance matrices of phylogenetic branch lengths between primate hosts and *p*-distance among parasite clades (calculated as described above) using the ape package [73] in the statistical programming language R (Development-Team 2008).

## Results

**Figure 1:**
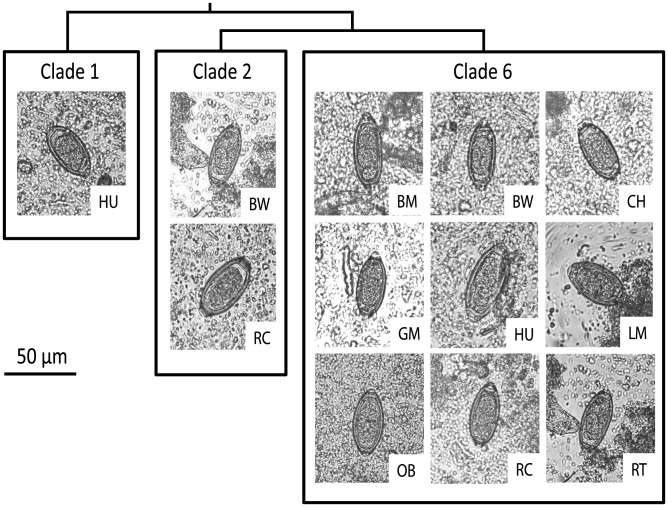
Representative eggs of *Trichuris* photographed at 40X objective magnification. *Trichuris* **eggs were identified in thin smears of sedimented feces from infected hosts.** Images demonstrate considerable morphological variation in egg size and shape (50–76 µm in length, 26–30 µm in width), although differences in the ratio of length to width among parasite clades and among host species were not significant (Kruskall-Wallis tests, *P*>0.05). The cladogram on the top of the figure is a simplified version of the phylogenetic tree shown in Figure 2 and represents the relative relatedness of *Trichuris* clades. Host species abbreviations follow Table 2.

We collected 282 samples from primates and 36 samples from humans, for a total of 318 samples. Of these, microscopy classified 104 samples as *Trichuris*-positive, making the community-wide prevalence of infection by microscopy 32.7% (Table 1). Eggs varied considerably in length (50–76 µm), and width (26–30 µm), but length-to-width ratios did not differ significantly among parasite clades (see below) or host species (Kruskall-Wallis test, *P*>0.05; Figure 1).

**Table 1:** Prevalence of *Trichuris* in nine primate (including human) hosts in and near Kibale National Park, Uganda based on microscopy and PCR of ITS1 and ITS 2 rDNA genes.

		Total No. *Trichuris* Positive		Prevalence (95% CI)		No. Sequenced	
*Species*	*N*	*Microscopy*	*PCR*	*Microscopy*	*PCR*	*ITS1*	*ITS2*
BM (Blue monkey)	33	9	9	27.3 (15–44)	27.3 (15–44)	5	5
BW (Black-and-white colobus)	37	12	13	32.4 (20–49)	35.1 (22–51)	9	9
CH (Chimpanzee)	30	4	4	13.3 (5–30)	13.3 (5–30)	3	1
GM (Gray-cheeked mangabey)	42	6	6	14.3 (6–28)	14.3 (6–28)	4	1
HU (Human)	36	8	11	22.2 (11–38)	30.6 (18–47)	9	4
LM (L'hoest monkey)	8	5	5	62.5 (30–87)	62.5 (30–87)	4	2
OB (Olive baboon)	27	24	24	88.9 (71–97)	88.9 (71–97)	8	3
RC (Red colobus)	64	15	15	23.4 (15–35)	23.4 (15–35)	9	5
RT (Red-tailed guenon)	41	21	22	51.2 (36–66)	53.7 (39–68)	11	5
TOTAL	318	104	108	32.7 (28–38)	34.0 (29–39)	62	35

PCR of ITS 1 and ITS 2 generated single, clear bands of expected size. PCR of ITS 1 and 2 generated identical results and were therefore considered together for the purposes of evaluating the diagnostic performance of PCR. PCR correctly classified all samples that were positive for *Trichuris* by microscopy. In addition, PCR classified five samples as positive for *Trichuris* that were negative by microscopy. Thus, the sensitivity of our new PCR assay was 100% (95% C.I. 96.5%–100.0%) and the specificity was 97.7% (95% C.I. 94.6%–99.2%), suggesting that our new PCR assays of ITS 1 and 2 are both highly accurate.

Prevalence varied significantly by species, (χ^2^ = 62.99, df = 8, *P*<0.0001), with chimpanzees (13.3%) and grey-cheeked mangabeys (14.3%) having the lowest prevalence, and olive baboons (88.9%) the highest (Table 1).

Of the 108 positive samples, 74 samples were selected for sequencing to represent the widest possible range of host species and, to the greatest extent possible, to equalize sequencing effort among host species. Because preliminary results indicated that ITS 1 provided greater phylogenetic resolution than ITS 2, 62 sequences for ITS 1 and 35 sequences for ITS 2 were ultimately generated (Supplementary Table S1). In samples where both ITS 1 and ITS 2 sequences were generated, sequences were concatenated and gaps were coded as missing data. The final alignment length of Gblock treated and concatenated ITS 1 and ITS 2 sequences was 1083 characters.

**Figure 2:**
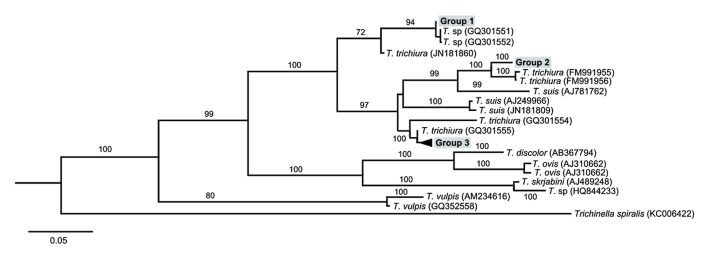
Bayesian phylogenetic tree of *Trichuris* based on concatenated ITS 1 and ITS 2 rDNA sequences. 18S, ITS 1 (895 bp), and 5.8S, ITS 2, 28S (486 bp) sequences were concatenated, and regions of ambiguous alignment removed in Gblocks. Phylogenetic relationships were inferred using MrBayes, with newly generated sequences clustering in three groups: Group 1 (2 human samples), Group 2 (4 black-and-white colobus and 1 red colobus samples), and Group 3 (7 blue monkey, 9 black-and-white colobus, 4 chimpanzee, 8 human, 4 grey-cheeked mangabey, 4 l'hoest monkey, 8 olive baboon, 10 red colobus, and 12 red-tailed guenon samples). Posterior clade probabilities are shown next to branches. Reference sequences (*T. trichiura*, *T suis*) and outgroups (*T. discolor*, *T. ovis*, *T. skrjabini*, *T. sp*, *T. vulpis* and *Trichinella spiralis*) are italicized, with GenBank accession numbers included in parenthesis. Scale bar indicates nucleotide substitutions per site.

Phylogenetic analysis resolved *Trichuris* into three groups, which, for convenience, we designate Groups 1, 2 and 3 in Figure 2. Group 1 contained two samples from humans that were 98.2% identical to each other and that most closely matched published sequences from Chacma baboons (*Papio hamadryas ursinus*) from South Africa (Genbank accession numbers GQ301551-2 [27]. This clade, along with a sequences from humans in Uganda [23] (Genbank accession numbers JN181837, JN181845), are sister to the *Trichuris* in-group and are the most genetically divergent lineage, sharing between 71.7% and 88.1% nucleotide similarity with other *T. trichiura* clades (Figure 2). Group 2 contained sequences from four black-and-white colobus and one red colobus that shared 100% nucleotide identity, and were most closely related to published *Trichuris* sequences from another subspecies of black-and-white colobus (*Colobus guereza kikuyuensis*) and yellow-cheeked gibbons (*Nomascus gabriellae*) from a zoo in Spain [22]. Finally, Group 3 contained 59 sequences from all seven species of primate host and eight humans sampled in this study. This group shared 99.3% nucleotide sequence identity and clustered most closely with published sequences from humans in Cameroon (accession number GQ301555), and more distantly with Chacma baboons in South Africa from the same study (accession number (GQ301554) [27]. All three sequences representing *T. suis* clustered within the *T. trichiura* species complex, and were most distinct (excluding outgroups) from Group 1 (66.9% nucleotide similarity), and most similar to Group 2 (88.5% nucleotide similarity).

Samples from human hosts identified in this study fell within Groups 1 and 3. Human-derived *Trichuris* sequences were most similar to those from grey-cheeked mangabey (95.2% similarity) and chimpanzees (95.1% identity), and most dissimilar to those of black-and-white colobus (91.2% identity; Table 2). When within-group sequence variation was held constant in PhiPT analysis, sequences from black-and-white colobus and olive baboons were significantly different, but sequences from other species pairs were not (Table 2). Mantel tests comparing host phylogeny and parasite *p*-distances between clades were not significant (Z-statistic = 43.37, *p* = 0.305). AMOVA revealed that 98% of *Trichuris* sequence-level variation was contained within host species, with only 2% of sequence-level variation apportioned between host species.

**Table 2:** Genetic differences between lineages of *Trichuris* from different host species.

	BM	BW	CH	LM	OB	GM	RC	RT	HU
**BM**	***0.001***	0.000	0.000	0.000	0.214	0.127	0.000	0.040	0.053
**BW**	0.025 (0.003)	***0.030***	0.032	0.052	0.227*	0.157	0.013	0.118	0.105
**CH**	0.002 (0.001)	0.032 (0.004)	***0.002***	0.000	0.000	0.000	0.000	0.000	0.000
**LM**	0.001 (0.000)	0.033 (0.004)	0.001 (0.001)	***0.000***	0.000	0.000	0.000	0.000	0.000
**OB**	0.007 (0.002)	0.041 (0.004)	0.009 (0.002)	0.008 (0.002)	***0.013***	0.000	0.034	0.000	0.000
**GM**	0.001 (0.000)	0.037 (0.004)	0.002 (0.001)	0.001 (0.000)	0.009 (0.002)	***0.001***	0.000	0.000	0.000
**RC**	0.008 (0.001)	0.030 (0.003)	0.010 (0.001)	0.009 (0.001)	0.017 (0.002)	0.010 (0.001)	***0.017***	0.000	0.000
**RT**	0.001 (0.000)	0.033 (0.004)	0.002 (0.001)	0.001 (0.000)	0.009 (0.002)	0.002 (0.000)	0.010 (0.001)	***0.002***	0.000
**HU**	0.063 (0.040)	0.088 (0.043)	0.049 (0.038)	0.053 (0.004)	0.058 (0.004)	0.048 (0.004)	0.061 (0.034)	0.052 (0.012)	***0.090***

Below diagonal: pairwise nucleotide differences per site averaged across all sequence pairs, with standard errors (calculated from 1000 bootstrap replicates) in parentheses. Above diagonal: *Trichuris* lineage differentiation between hosts (PhiPT; an analog of F_ST_), with probability values based on 999 permutations. Significant values (P<0.05) generated from 999 permutations are indicated with asterisks. Within diagonal: within-group nucleotide substitutions per site between all sequences within a host species. Host species abbreviations are: BM  =  Blue monkey, BW  =  Black-and-white colobus, CH  =  Chimpanzee, LM  =  L'hoest monkey, OB  =  Olive baboon, RC  =  Red colobus, RT  =  Red-tailed guenon, and HU  =  Human.

## Discussion

We investigated the taxonomy and phylogenetic structure of the whipworm genus *Trichuris* in a wild primate community and a nearby human population in Uganda. The overall prevalence of infection was 34%, but this varied significantly among host species, with the lowest prevalence in chimpanzees (13.3%) and the highest prevalence in olive baboons (88.9%). Research in Gombe National Park, Tanzania, where these two species also overlap, found similar results, with chimpanzees having 5% infection prevalence and baboons 66% [74]. Averaging across sites in Tanzania and Senegal, another study found prevalences of 4.5% and 35% in chimpanzees and baboons, respectively. Interestingly, *Trichuris* is one of the few parasites with consistently higher prevalence in baboons than in chimpanzees [75]. In an attempt to explain interspecific differences in prevalence, we conducted a phylogenetic-least-squared regression to explore correlations between host traits (terrestriality, home range, group size, time spent in polyspecific associations, body mass, and daily travel distance) and prevalence (not shown), but found no significant relationships. It therefore remains unclear why *Trichuris* prevalence varies significantly among sympatric primate hosts.

In humans, our results indicate a prevalence of 30.6% by PCR. Previous research in Uganda has estimated prevalence to be between 12.9 and 28% using microscopy; however, this is among school-aged children, where the frequency of *Trichuris* infection is high [76], [77]. Our estimate of 30.6% infection in a human community containing individuals of multiple ages suggests that this region of Uganda has a generally high rate of infection. Poor access to latrines, earthen flooring in houses, and large family sizes are likely contributing factors [78], although improved accuracy of our methods relative to others may also help explain this difference.

Our phylogenetic analysis revealed that *Trichuris* sequences from the Kibale primate community and neighboring human population sorted into three groups. Group 1 contained two sequences from humans and clustered closely with sequences derived from Chacma baboons [27]. Interestingly, these sequences were designated as part of the most phylogenetically distinct *T. trichiura* clade, most distant from *T. suis* [27]. The authors of these sequences therefore refrained from designating this clade *T. trichiura* [27]. Our results support the conclusion that this *Trichuris* clade represents a separate species, since our phylogenetic analysis placed Group 1 and associated published reference sequences as sister to all other *Trichuris* in-groups. Our *p*-distance analyses similarly estimate the maximum dissimilarity between Group 1 (and associated published reference sequences) and all other in-groups to be 33.1%, which is nearly twice that between previously described sequences of *T. trichiura* and *T. suis*, which are recognised as taxonomically distinct species.

Group 1 sequences and GQ301551-2 were sister to sequences that were part of study that sought to identify genetic similarity between *T. trichiura* and *T. suis* derived from humans and pigs living in close proximity [23]. In the latter study, two distinct genotypes of human-derived *Trichuris* were defined, which they designated type 1 and type 2 [23]. Our sequences cluster with their type 1 genotype (represented by JN181860 in Figure 2), the clade more distantly related to *T. suis*. Despite screening the entire diurnal primate community, we detected Group 1 only in humans. However, a similar genotype has been found elsewhere in baboons [27], suggesting that the Group 1 lineage may have a broader host range than documented in our study, perhaps indicating a potential for infrequent cross-species transmission.

In contrast to *Trichuris* Group 1, we detected *Trichuris* Group 3 in every host species sampled, including humans. This result suggests that Group 3, including published reference sequences, represents a multi-host lineage capable of infecting multiple primate hosts, including humans. Our population analyses support these results, in that only 2% of overall *Trichuris* genetic variation is apportioned between host species.

Group 2, containing sequences derived from black-and-white colobus and red colobus, also appears to be a distinct lineage. This clade is most closely related to *T. trichiura* from other primates, namely gibbons and another subspecies of black-and-white colobus [22]. This *Trichuris* lineage may have an intermediate host range compared to Groups 1 and 3, given that all samples save one were derived from colobus monkeys. The one sample that was not derived from colobines (gibbon) was collected from a zoo, and may therefore reflect transmission outside of a natural setting. Additional sampling and sequencing would help clarify the host range of this *Trichuris* taxon.

We note that rDNA occurs in multiple copies, and this study does not attempt to quantify intraspecific variation or mixed lineage infections. Our data therefore reflect a minimum conservative estimate of parasite genetic variation. Similarly, we note that our data could reflect variation among paralogs within and among infections, although we found no direct evidence for this. However, such intra-individual diversity is almost certainly lower than diversity between hosts, such that it is unlikely to have confounded the overall patterns we describe.

In our study area, several primates frequently raid crops, with the most common offenders being baboons, red tailed guenons, and chimpanzees [39], [79], [80]. Such interactions facilitate the transmission of gastrointestinal bacteria, protozoa and helminths in the Kibale system [43], [49], [52], [53], [81], [82]. Although these interactions make cross-species transmission ecologically plausible, it remains unclear why one *Trichuris* lineage appears able to cross species boundaries with apparent ease, yet another other clades show host affinity (Group 1).

In conclusion, our phylogenetic analysis suggests that *Trichuris* is not a single species, but a species complex (see also Nissen et al. [23] and Liu et al. [29]) of co-circulating clades that includes *T. suis.* Despite being sympatric, different clades appear to have different host affinity. Group 1 was specific to humans in our study, Group 2 has an intermediate host range, and Group 3 appears capable of infecting all primates sampled, including humans. While our analyses do not indicate whether Group 3 *Trichuris* is transmitted from primates to humans or *vice versa*, they do show that certain lineages within the *Trichuris* taxonomic complex should be considered multi-host pathogens, at least within the order Primates. Our results also demonstrate that *Trichuris* is among the 20% of helminths capable of cross-infecting primates and humans. Taxonomic and epidemiological studies of other soil-transmitted helminths in wild primates, many of which cause “neglected” tropical diseases [83], may reveal yet more helminth taxa to be multi-host pathogens. If so, this would challenge past assumptions about the host specificity of helminth parasites while raising new concerns about global human and animal health.

## Supporting Information

Table S1 Sequence dataset. Putative species: all from genus *Trichuris*. Location: KNP  =  Kibale National Park. DGP  =  Da Gamma Park. GOB  =  Groot Olifant Bos. CP  =  Cape Peninsula. Date: date of sample collection. Acc. No.: Genbank accession number for each respective gene, where bolded accession numbers indicate sequences generated in this study. [P]  =  partial gene sequence. [C]  =  complete gene sequence. Putative species marked with “*” indicates sequences which our analysis suggests belong to *Trichuris* species different from those identified in published GenBank entries. Accession numbers JN181833, JN181845, and JN181860 are listed as *T. trichiura* in GenBank but are identified as *T*. sp here. Accession numbers GQ301551-3 are listed as *T*. sp in Genbank, but are identified as *T. trichiura* here.
(PDF)
