# Supplementary material for: Hidden Population Structure and Cross-species Transmission of Whipworms (Trichuris sp.) in Humans and Non-human Primates in Uganda
Source: PLoS Negl Trop Dis. 2014 Oct 23;8(10):e3256. doi: 10.1371/journal.pntd.0003256 (PMC4207677; doi:10.1371/journal.pntd.0003256)
Supplement: Table S1 — Sequence dataset. Putative species: all from genus Trichuris. Location: KNP = Kibale National Park. DGP = Da Gamma Park. GOB = Groot Olifant Bos. CP = Cape Peninsula. Date: date of sample collection. Acc. No.: Genbank accession number for each respective gene, where bolded accession numbers indicate sequences generated in this study. [P] = partial gene sequence. [C] = complete gene sequence. Putative species marked with “*” indicates sequences which our analysis suggests belong to Trichuris species different from those identified in published GenBank entries. Accession numbers JN181833, JN181845, and JN181860 are listed as T. trichiura in GenBank but are identified as T. sp here. Accession numbers GQ301551-3 are listed as T. sp in Genbank, but are identified as T. trichiura here. (PDF) [file pntd.0003256.s001.pdf]

HOST SPECIES	SAMPLE ID	PUTATIVE SPECIES	LOCATION	GPS NORTHING, EASTING	DATE (D/M/Y)	ACC. No. 18S	ACC. No. ITS1	ACC. No. 5.8S	ACC. No. ITS2	ACC. No. 28S
**In-group**										
Cercopithecus ascanius	RT005	*T. trichiura*	KNP, Uganda	0205673, 0062388	30/05/11	**KJ588082 [P]**	**KJ588082 [P]**	-	-	-
	RT014	*T. trichiura*	KNP, Uganda	0205777, 0062630	30/05/11	**KJ588117 [P]**	**KJ588117 [P]**	**KJ588150 [P]**	**KJ588150 [C]**	**KJ588150 [P]**
	RT018	*T. trichiura*	KNP, Uganda	0205784, 0062342	31/05/11	**KJ588081 [P]**	**KJ588081 [P]**	-	-	-
	RT043	*T. trichiura*	KNP, Uganda	0207054, 0061321	01/06/11	**KJ588097 [P]**	**KJ588097 [P]**	**KJ588135 [P]**	**KJ588135 [C]**	**KJ588135 [P]**
	RT046	*T. trichiura*	KNP, Uganda	0207072, 0061402	01/06/11	**KJ588123 [P]**	**KJ588123 [P]**	**KJ588145 [P]**	**KJ588145 [C]**	**KJ588145 [P]**
	RT125	*T. trichiura*	KNP, Uganda	0205476, 0061343	23/06/11	**KJ588079 [P]**	**KJ588071 [P]**	-	-	-
	RT127	*T. trichiura*	KNP, Uganda	0205478, 0061349	23/06/11	-	-	**KJ588153 [P]**	**KJ588153 [C]**	**KJ588153 [P]**
	RT147	*T. trichiura*	KNP, Uganda	0205781, 0062416	25/06/11	**KJ588073 [P]**	**KJ588071 [P]**	-	-	-
	RT150	*T. trichiura*	KNP, Uganda	0205755, 0062459	25/06/11	**KJ588106 [P]**	**KJ588106 [P]**	**KJ588148 [P]**	**KJ588148 [C]**	**KJ588148 [P]**
	RT151	*T. trichiura*	KNP, Uganda	0205729, 0062435	25/06/11	**KJ588119 [P]**	**KJ588119 [P]**	-	-	-
	RT152	*T. trichiura*	KNP, Uganda	0205886, 0062435	25/06/11	**KJ588091 [P]**	**KJ588091 [P]**	-	-	-
	RT217	*T. trichiura*	KNP, Uganda	0206364, 0061892	06/07/11	**KJ588112 [P]**	**KJ588112 [P]**	-	-	-
Cercopithecus l'hoesti	LM252	*T. trichiura*	KNP, Uganda	0203567, 0064093	16/07/11	**KJ588102 [P]**	**KJ588102 [P]**	**KJ588164 [P]**	**KJ588164 [C]**	**KJ588164 [P]**
	LM253	*T. trichiura*	KNP, Uganda	0203567, 0064093	16/07/11	**KJ588109 [P]**	**KJ588109 [P]**	-	-	-
	LM261	*T. trichiura*	KNP, Uganda	0205844, 0061342	19/07/11	**KJ588116 [P]**	**KJ588116 [P]**	**KJ588163 [P]**	**KJ588163 [C]**	**KJ588163 [P]**
	LM262	*T. trichiura*	KNP, Uganda	0205904, 0061210	19/07/11	**KJ588118 [P]**	**KJ588118 [P]**	-	-	-
Cercopithecus mitis	BM175	*T. trichiura*	KNP, Uganda	0206768, 0064255	30/06/11	**KJ588120 [P]**	**KJ588120 [P]**	**KJ588141 [P]**	**KJ588141 [C]**	**KJ588141 [P]**
	BM194	*T. trichiura*	KNP, Uganda	0206399, 0061807	04/07/11	**KJ588110 [P]**	**KJ588110 [P]**	**KJ588165 [P]**	**KJ588165 [C]**	**KJ588165 [P]**
	BM211	*T. trichiura*	KNP, Uganda	0206349, 0061859	06/07/11	**KJ588113 [P]**	**KJ588113 [P]**	-	-	-
	BM213	*T. trichiura*	KNP, Uganda	0206351, 0061863	06/07/11	**KJ588115 [P]**	**KJ588115 [P]**	-	-	-
	BM272	*T. trichiura*	KNP, Uganda	0206479, 0064024	21/07/11	**KJ588104 [P]**	**KJ588104 [P]**	**KJ588156 [P]**	**KJ588156 [C]**	**KJ588156 [P]**
	BM280	*T. trichiura*	KNP, Uganda	0206153, 0061338	22/07/11	-	-	**KJ588138 [P]**	**KJ588138 [C]**	**KJ588138 [P]**
	BM281	*T. trichiura*	KNP, Uganda	0206122, 0061428	22/07/11	-	-	**KJ588143 [P]**	**KJ588143 [C]**	**KJ588143 [P]**
Colobus guereza	BW072	*T. trichiura*	KNP, Uganda	0206251, 0062623	03/06/11	**KJ588076 [P]**	**KJ588076 [P]**	**KJ588167 [P]**	**KJ588167 [C]**	**KJ588167 [P]**
	BW075	*T. trichiura*	KNP, Uganda	0206263, 0062633	03/06/11	**KJ588085 [P]**	**KJ588085 [P]**	**KJ588166 [P]**	**KJ588166 [C]**	**KJ588166 [P]**
	BW076	*T. trichiura*	KNP, Uganda	0206251, 0062621	03/06/11	**KJ588086 [P]**	**KJ588086 [P]**	**KJ588157 [P]**	**KJ588157 [C]**	**KJ588157 [P]**
	BW108	*T. trichiura*	KNP, Uganda	0206182, 0061352	21/06/11	**KJ588087 [P]**	**KJ588087 [P]**	**KJ588158 [P]**	**KJ588158 [C]**	**KJ588158 [P]**
	BW114	*T. trichiura*	KNP, Uganda	0205490, 0061411	22/06/11	**KJ588129 [P]**	**KJ588129 [P]**	-	-	-
	BW115	*T. trichiura*	KNP, Uganda	0205464, 0061430	22/06/11	-	-	**KJ588136 [P]**	**KJ588136 [C]**	**KJ588136 [P]**
	BW116	*T. trichiura*	KNP, Uganda	0205493, 0061408	22/06/11	**KJ588131 [P]**	**KJ588131 [P]**	-	-	-
	BW117	*T. trichiura*	KNP, Uganda	0205480, 0061437	22/06/11	-	-	**KJ588149 [P]**	**KJ588149 [C]**	**KJ588149 [P]**
	BW118	*T. trichiura*	KNP, Uganda	0205488, 0061411	22/06/11	-	-	**KJ588161 [P]**	**KJ588161 [C]**	**KJ588161 [P]**
	BW153	*T. trichiura*	KNP, Uganda	0206167, 0064497	27/06/11	**KJ588077 [P]**	**KJ588077 [P]**	**KJ588139 [P]**	**KJ588139 [C]**	**KJ588139 [P]**
	BW154	*T. trichiura*	KNP, Uganda	0206073, 0064514	27/06/11	-	-	**KJ588146 [P]**	**KJ588146 [C]**	**KJ588146 [P]**
	BW266	*T. trichiura*	KNP, Uganda	0207114, 0064725	20/07/11	**KJ588130 [P]**	**KJ588130 [P]**	-	-	-
	BW268	*T. trichiura*	KNP, Uganda	0207148, 0064634	20/07/11	**KJ588132 [P]**	**KJ588132 [P]**	-	-	-
	FM991956	*T. trichiura*	Málaga, Spain	-	-	-	FM991956	FM991956 [C]	FM991956	-

Homo sapiens	HU009	*T. trichiura*	Kabarole, Uganda	0205748, 0062482	28/07/11	**KJ588121 [P]**	**KJ588121 [P]**	-	-	-
	HU012	*T.* sp.	Kabarole, Uganda	0205120, 0062531	28/07/11	**KJ588071 [P]**	**KJ588071 [P]**	**KJ588133 [P]**	**KJ588133 [C]**	**KJ588133 [P]**
	HU014	*T. trichiura*	Kabarole, Uganda	0205018, 0062448	01/08/11	**KJ588124 [P]**	**KJ588124 [P]**	-	-	-
	HU016	*T.* sp.	Kabarole, Uganda	0204022, 0060141	01/08/11	**KJ588072 [P]**	**KJ588072 [P]**	-	-	-
	HU026	*T. trichiura*	Kabarole, Uganda	0204104, 0063292	02/08/11	**KJ588090 [P]**	**KJ588090 [P]**	-	-	-
	HU027	*T. trichiura*	Kabarole, Uganda	0204104, 0063292	02/08/11	-	-	**KJ588151 [P]**	**KJ588151 [C]**	**KJ588151 [P]**
	HU030	*T. trichiura*	Kabarole, Uganda	0204068, 0063089	05/08/11	**KJ588088 [P]**	**KJ588088 [P]**	**KJ588134 [P]**	**KJ588134 [C]**	**KJ588134 [P]**
	HU035	*T. trichiura*	Kabarole, Uganda	0204191, 0063178	05/08/11	**KJ588100 [P]**	**KJ588100 [P]**	-	-	-
	HU036	*T. trichiura*	Kabarole, Uganda	0204191, 0063178	05/08/11	**KJ588089 [P]**	**KJ588089 [P]**	**KJ588159 [P]**	**KJ588159 [C]**	**KJ588159 [P]**
	HU037	*T. trichiura*	Kabarole, Uganda	0204314, 0063027	05/08/11	**KJ588075 [P]**	**KJ588075 [P]**	-	-	-
	GQ301555	*T. trichiura*	Kumba, Cameroon	-	-/-/00	-	GQ301555 [P]	GQ301555 [C]	GQ301555 [P]	-
	JN181833	*T.* sp*	Kabale, Uganda	-	-/-/08	-	-	-	JN181833 [C]	-
	JN181845	*T.* sp*	Kabale, Uganda	-	-/-/08	-	-	-	JN181845 [C]	-
	JN181860	*T.* sp*	Kabale, Uganda	-	-/-/08	-	-	-	JN181860 [C]	-
Lophocebus albigena	GM070	*T. trichiura*	KNP, Uganda	0206835, 0061683	02/06/11	**KJ588096 [P]**	**KJ588096 [P]**	-	-	-
	GM095	*T. trichiura*	KNP, Uganda	0207038, 0060627	07/06/11	**KJ588107 [P]**	**KJ588107 [P]**	-	-	-
	GM249	*T. trichiura*	KNP, Uganda	0207136, 0060961	14/07/11	**KJ588101 [P]**	**KJ588101 [P]**	-	-	-
	GM250	*T. trichiura*	KNP, Uganda	0205370, 0062918	15/07/11	**KJ588111 [P]**	**KJ588111 [P]**	**KJ588147 [P]**	**KJ588147 [C]**	**KJ588147 [P]**
Nomascus gabriellae	FM991955	*T. trichiura*	Málaga, Spain	-	-	-	FM991955	FM991955 [C]	FM991955	-
Pan troglodytes	CH236	*T. trichiura*	KNP, Uganda	-	08/07/11	-	-	**KJ588160 [P]**	**KJ588160 [C]**	**KJ588160 [P]**
	CH239	*T. trichiura*	KNP, Uganda	-	10/07/11	**KJ588094 [P]**	**KJ588094 [P]**	-	-	-
	CH240	*T. trichiura*	KNP, Uganda	-	10/07/11	**KJ588103 [P]**	**KJ588103 [P]**	-	-	-
	CH242	*T. trichiura*	KNP, Uganda	-	10/07/11	**KJ588078 [P]**	**KJ588078 [P]**	-	-	-
Papio anubis	OB078	*T. trichiura*	KNP, Uganda	0205740, 0061953	04/06/11	**KJ588084 [P]**	**KJ588084 [P]**	-	-	-
	OB080	*T. trichiura*	KNP, Uganda	0205779, 0061996	04/06/11	**KJ588099 [P]**	**KJ588099 [P]**	**KJ588152 [P]**	**KJ588152 [C]**	**KJ588152 [P]**
	OB081	*T. trichiura*	KNP, Uganda	0205745, 0061963	04/06/11	**KJ588098 [P]**	**KJ588098 [P]**	-	-	-
	OB082	*T. trichiura*	KNP, Uganda	0205739, 0061954	04/06/11	**KJ588125 [P]**	**KJ588125 [P]**	**KJ588154 [P]**	**KJ588154 [C]**	**KJ588154 [P]**
	OB185	*T. trichiura*	KNP, Uganda	0205755, 0061972	05/06/11	**KJ588105 [P]**	**KJ588105 [P]**	-	-	-
	OB189	*T. trichiura*	KNP, Uganda	0206826, 0064528	01/07/11	**KJ588114 [P]**	**KJ588114 [P]**	-	-	-
	OB285	*T. trichiura*	KNP, Uganda	0206153, 0061338	22/07/11	**KJ588126 [P]**	**KJ588126 [P]**	**KJ588155 [P]**	**KJ588155 [C]**	**KJ588155 [P]**
	OB286	*T. trichiura*	KNP, Uganda	0206122, 0061428	22/07/11	**KJ588127 [P]**	**KJ588127 [P]**	-	-	-
Papio hamadryas	GQ301551	*T. trichiura**	DGP, CP, S. Africa	-	-/-/06-08	-	GQ301551 [P]	GQ301551 [C]	GQ301551 [P]	-
	GQ301552	*T. trichiura**	DGP, CP, S. Africa	-	-/-/06-08	-	GQ301552 [P]	GQ301552 [C]	GQ301552 [P]	-
	GQ301553	*T. trichiura**	DGP, CP, S. Africa	-	-/-/06-08	-	GQ301552 [P]	GQ301552 [C]	GQ301553 [P]	-
	GQ301554	*T. trichiura*	GOB, CP, S. Africa	-	-/-/06-08	-	GQ301554 [P]	GQ301553 [C]	GQ301554 [P]	-
Procolobus rufomitratus	RC009	*T. trichiura*	KNP, Uganda	0205684, 0062397	30/05/11	**KJ588083 [P]**	**KJ588083 [P]**	**KJ588137 [P]**	**KJ588137 [C]**	**KJ588137 [P]**
	RC010	*T. trichiura*	KNP, Uganda	0205677, 0062380	30/05/11	**KJ588128 [P]**	**KJ588128 [P]**	-	-	-
	RC012	*T. trichiura*	KNP, Uganda	0205657, 0062357	30/05/11	-	-	**KJ588140 [P]**	**KJ588140 [C]**	**KJ588140 [P]**
	RC017	*T. trichiura*	KNP, Uganda	0205784, 0062342	30/05/11	-	-	**KJ588142 [P]**	**KJ588142 [C]**	**KJ588142 [P]**
	RC142	*T. trichiura*	KNP, Uganda	0206644, 0062131	24/06/11	**KJ588093 [P]**	**KJ588093 [P]**	-	-	-

	RC143	*T. trichiura*	KNP, Uganda	0206640, 0062129	24/06/11	**KJ588108 [P]**	**KJ588108 [P]**	-	-	-
	RC161	*T. trichiura*	KNP, Uganda	0207114, 0064725	29/06/11	**KJ588080 [P]**	**KJ588080 [P]**	-	-	-
	RC182	*T. trichiura*	KNP, Uganda	0206205, 0064843	29/06/11	-	-	**KJ588144 [P]**	**KJ588144 [C]**	**KJ588144 [P]**
	RC184	*T. trichiura*	KNP, Uganda	0206994, 0064010	01/07/11	**KJ588095 [P]**	**KJ588095 [P]**	-	-	-
	RC218	*T. trichiura*	KNP, Uganda	0206194, 0062072	06/07/11	**KJ588092 [P]**	**KJ588092 [P]**	-	-	-
	RC222	*T. trichiura*	KNP, Uganda	0206195, 0062075	06/07/11	**KJ588074 [P]**	**KJ588074 [P]**	-	-	-
	RC223	*T. trichiura*	KNP, Uganda	0206196, 0062075	06/07/11	**KJ588122 [P]**	**KJ588122 [P]**	**KJ588162 [P]**	**KJ588162 [C]**	**KJ588162 [P]**
Sus scrofa	JN181810	*T. suis*	Tanzania	-	-/08/08	-	-	-	JN181810 [C]	-
	AJ249966	T. suis	Andalucia, Spain	-	-	-	-	-	AJ249966 [C]	-
	AJ781762	T. suis	Spain	-	-	-	AJ781762 [C]	-	-	-
	AJ783398	*T. suis*	Spain	-	-	-	AJ783398 [C]	-	-	-
**Out-group**										
Canis lupus	GQ352558	*T. vulpis*	Thailand	-	-	GQ352558 [P]	GQ352558 [C]	GQ352558 [P]	-	-
	AM234616	*T. vulpis*	Seville, Spain	-	-	-	AM234616 [P]	AM234616 [C]	AM234616 [P]	-
Capra hircus	AJ489248	*T. skrjabini*	Seville, Spain	-	-	-	AJ489248 [P]	AJ489249 [C]	AJ489248 [C]	-
Capricornis crispus	AB367794	*T. discolor*	Iwate, Japan	-	-	-	AB367794 [C]	AB367794 [C]	AB367794 [C]	-
Ovis aries	HQ844233	*T.* sp	China	-	-	HQ844233 [P]	HQ844233 [C]	HQ844233 [C]	HQ844233 [C]	HQ844233 [P]
Unknown	AJ238220	*T. ovis*	Andalucia, Spain	-	-	-	-	-	AJ238220 [C]	-
	AJ310662	*T. ovis*	Spain	-	-	-	AJ310662 [C]	-	-	-
